# Multi-Indication Carbamazepine and the Risk of Severe Cutaneous Adverse Drug Reactions in Korean Elderly Patients: A Korean Health Insurance Data-Based Study

**DOI:** 10.1371/journal.pone.0083849

**Published:** 2013-12-31

**Authors:** Ji Young Kim, Joongyub Lee, Young-Jin Ko, Ju-Young Shin, Sun-Young Jung, Nam-Kyong Choi, Byung-Joo Park

**Affiliations:** 1 Department of Preventive Medicine, Seoul National University College of Medicine, Seoul, Republic of Korea; 2 Medical Research Collaborating Center, Seoul National University Hospital, Seoul, Republic of Korea; 3 Korea Institute of Drug Safety and Risk Management, Seoul, Republic of Korea; Universidad de Valladolid, Spain

## Abstract

**Objective:**

To evaluate the risk of severe cutaneous adverse drug reactions (SCAR) after exposure to multi-indication antiepileptic drugs for in Korean elderly patients.

**Methods:**

We used a nationwide database from the Korean Health Insurance Review and Assessment Service claims constructed for the monitoring of drug utilization among the entire Korean elderly population from January 2005 to June 2006. We identified cases of SCARs among inpatients aged ≥65 years and those newly diagnosed with erythema multiforme according to the International Classification of Diseases, 10th revision code (L51). Each case was matched to four controls for gender, age, and the first hospitalization date as the index date. The use of carbamazepine, gabapentin, lamotrigine, topiramate, phenobarbital, phenytoin, and valproate during a 60-day period before the index date was compared. A conditional logistic regression analysis was performed to calculate the odds ratios (OR) and 95% confidence intervals (CI) of SCARs for antiepileptic drug.

**Results:**

We identified 286 cases of SCAR and 1,144 matched controls. Among the 25 patients who were prescribed antiepileptic drugs within 60 days of the index date. There were 11 cases (3.8%) of severe ocular manifestations, and most elderly patients were first-time or short-term users of antiepileptic drugs. Among the 10 cases of carbamazepine use, only 2 cases were prescribed carbamazepine for seizure. All antiepileptic drugs were associated with an increased SCAR risk (adjusted OR = 3.42, 95% CI: 1.75–6.63). The SCAR risk was highest in patients treated with carbamazepine (adjusted OR = 10.39, 95% CI: 2.64–40.86, for multi-indication; adjusted OR = 6.84, 95% CI: 1.55–30.10, for neuropathic pain).

**Conclusion:**

Carbamazepine use was associated with a nearly 10-fold increase in severe cutaneous drug reactions in Korean elderly patients. This association was consistently high with SCAR patients who received carbamazepine for neuropathic pain.

## Introduction

Antiepileptic drugs including carbamazepine, valproate, phenytoin, and phenobarbital consistently increase the risk of severe cutaneous adverse drug reactions (SCARs) such as Stevens-Johnson syndrome (SJS) and toxic epidermal necrolysis (TEN) [1]–[3]. Nevertheless, the use of antiepileptic drugs has steadily increased in elderly patients. Antiepileptic drugs are prescribed for the treatment of diverse conditions including migraines, mood disorders, neuropathic pain, and epilepsy [4]–[6]. The use of antiepileptic drugs peaked in those aged 80–84 years in Australia [4] and according to the Norwegian Prescription Database constructed between 2004 and 2007, 24.5% of antiepileptic drugs users were elderly people (aged 60–102 years) [5]. Neuropathic pain such as post-herpes neuralgia, diabetic neuropathy, and central post-stroke pain, as well as post-stroke seizure and epilepsy are the most common reasons for antiepileptic drug use in the elderly [7], [8]. Moreover, the clearance rate of most antiepileptic drugs in the elderly has decreased by approximately 20–40% [9].

Elderly patients are more vulnerable to adverse drug reactions than younger patients, because of multi-morbidity and polypharmacy [10], [11]. These factors are important cause of hospital admissions in the elderly [12], [13]. One study found that inpatients with adverse drug reactions (ADRs) were significantly older (median age, 76 years) than patients without ADRs (median age, 66 years) [13]. Most of the cutaneous ADRs are not life threatening, but they can be a major cause of poor quality of life among the elderly [14]. SJS and TEN have a high mortality rate of 20%–25%, and long-lasting sequelae including corneal ulcerations represent a considerable disease burden [15]. Additionally, the mortality in SJS and TEN patients increased with age [16]. Recently, a 1-year follow-up study of patients with SCARs reported that high in-hospital mortality and after-discharge deaths were associated with older age [17].

There are few recent data on the epidemiology of SCARs in the elderly. Previous studies on SCAR were conducted across all age groups in the West, but did not specifically focus on the elderly. A recent SCAR study using the National Health Insurance database in Taiwan focused on bipolar disorders in middle-aged patients (mean age, 41 years) [18].

To our knowledge, there are no previous large-scale epidemiological studies of antiepileptic drug-related SCARs on the Korean elderly. In 2007, a nationwide database was constructed for monitoring drug utilization among the elderly in Korea. The aim of this study was to evaluate the risk of SCARs after exposure to multi-indication antiepileptic drugs in Korean patients aged ≥65 years. We conducted a case-control study based on the Korean Health Insurance Review and Assessment Service (HIRA) database constructed from January 1, 2005, to June 30, 2006.

## Materials and Methods

### Data Source

We used the Korean HIRA database of elderly patients aged ≥65 years, which was constructed between January 1, 2005, and June 30, 2006. The database contained information on 4,159,305 elderly patients aged ≥65 years and 100,838,744 prescriptions. The HIRA database contained information on the patients' de-identified number, age, gender, institutional region, diagnosis as per the International Classification of Diseases 10th Revision code (ICD-10 code), prescribed drug names, formulas, doses, prescription duration, and expenses [19]. The Korean National Health Insurance (KNHI) program was initiated in 1977 and achieved universal coverage for the entire Korean population of approximately 50 million individuals by 1989 [20]. The HIRA provided de-identified patient claim data for the purpose of public health research. In the HIRA database, the positive predictive value (PPV) for the diagnosis of inpatients was 81.8% [21].

### Ethics Statement

This study was approved by the Institutional Review Board of Seoul National University College of Medicine/Seoul National University Hospital (E-1305-036-487).

### Definition of Case and Control Patients

Cases were defined as inpatients aged ≥65 years who newly diagnosed with erythema multiforme (ICD-10 code: L51) between April 1, 2005, and June 30, 2006 [18], [22]–[24]. The first hospitalization due to erythema multiforme was defined as the index date for the case. To identify incident cases of SCAR, we excluded patients with pre-existing erythema multiforme during the preceding 3 months, from January 1, 2005, to March 31, 2005. Among them, patients diagnosed prior to the index date with epidermolysis bullosa (Q81), Kawasaki's disease (Q81), pemphigus (L10), pemphigoid (L12), psoriasis (L40), staphylococcal scalded skin syndrome (L00), toxic shock syndrome (A48.3), vasculitis (L95), or graft-versus-host disease (T86.0), according to the exclusion criteria of the European Registry of Severe Cutaneous Adverse Reactions (RegiSCAR) study, were also excluded in order to increase the validity of SCAR diagnosis [17], [25]. Potential controls were selected among inpatients with no diagnosis of skin and subcutaneous tissue disease (L00–L99) during the study periods. The exclusion criteria in controls were also based on the same as the criteria used in the cases. The index date of control patients were defined as the date of hospital admission for treatment. For each incident case, four controls were matched by gender, age (±5 years), and index date (±5 days).

### Exposure Assessment

Patients who were prescribed at least one antiepileptic drug before the index date were defined as the antiepileptic drugs prescription group. Seven antiepileptic drugs – carbamazepine, gabapentin, lamotrigine, topiramate, phenobarbital, phenytoin, and valproate – were selected as the exposure agents. Patients who were simultaneously prescribed different antiepileptic drugs during the research period were classified into the combination-therapy group, and those who were prescribed only one medication were classified into the mono-therapy group. The exposure period was defined as the prescription of antiepileptic drugs within 60 days before the index date. Previous studies reported that the risk of SCARs increases within 8 weeks following the first use of such drugs [3], [18], [26]. To estimate the time delay between the beginning of antiepileptic drug use and the occurrence of SCARs, the latent period was defined as the duration between the date of the antiepileptic drug prescription and the index date. To evaluate the effect of short- or long-term use, the prescription duration was defined as the total period for which antiepileptic drugs were prescribed before the index date.

### Confounding Factors

Information on confounding factors was collected using ICD-10 codes for diagnoses and drug prescriptions in the HIRA database. Cancer (C00-C97), connective tissue/rheumatic diseases (M05, M06, M32-M 34, M31.5, M35.1, M35.3, M36.0), herpes virus infection (B00), Human immunodeficiency virus (HIV) infection (B20–B24), influenza (J11), histoplasmosis (B39), and mycoplasma pneumonia (J15.7), which are non-medication risk factors for SJS or TEN, were included [3], [26]. To adjust for the severity of accompanying diseases, Charlson Comorbidity Index scores were calculated [27]. In addition, bipolar disorder (F31), diseases of the spinal cord (G95), neuropathy (G56–G64, G99), trigeminal neuralgia (G50, G53), seizures (G40, G41, R56), cerebrovascular diseases (G45, G46, I60–I69, H34.0), diabetes mellitus (E10–E14), and renal diseases (N18, N19, N05.2–N05.7, N03.2–N03.7, Z49.0–Z49.2) were included as underlying diseases before the index date. Other concomitant medications such as acetate derivative non-steroidal anti-inflammatory drugs (NSAIDs), allopurinol, aminopenicillins, cephalosporin, imidazole, macrolide, oxicam NSAIDs, quinolones, sulfonamide, and tetracyclines, which are related to the risk of SCARs, were considered as confounding factors [28].

### Statistical Analysis

The general characteristics of patients, including age, gender, prescription of antiepileptic drugs, comorbidities, and Charlson Comorbidity Index scores, were analyzed. Categorical variables are presented as numbers and percentages and continuous variables are presented as means and standard deviations (SD). For the continuous variables, we used the Wilcoxon signed-ranks test to compare means. Comorbidities of cases and controls were compared using the McNemar test. For each incident case, four controls were matched by gender, age (±5 years) and index date (±5 days) using the Greedy method. Because controls were matched individually, conditional logistic regression was used to assess the risk of SCARs after use of antiepileptic drugs. If exposure rate of the antiepileptic drug in the controls is assumed to be 2% and the odds ratios (OR), 3.0 based on results of previous studies, then on taking into account the 1∶4 matched case-control study design, the appropriate number of study subjects was calculated to be 284 in the case group, which would yield a 80% statistical power and 5% significance. Univariate and multivariate conditional logistic regression analyses were performed to calculate the ORs and 95% confidence intervals (CI) of SCARs for each antiepileptic drug. For some antiepileptic drugs taken by few or no patients, we used the exact method for estimating ORs. Among comorbidities and co-medications previously established as risk factors, variables with *p*-values <0.2 in the univariate analysis were selected as potential confounders for the final multivariate model. Subgroup analyses were performed only for SJS (L51.1) or TEN (L51.2) with the four-digit ICD-10 codes to assess the validated patients with SCAR. Latent periods and prescription durations were calculated using both medians (range) and means (SD). All *p*-values of <0.05 were considered statistically significant, and all tests were two-tailed. All data were analyzed using SAS 9.3 (SAS Institute Inc., Cary, NC, USA).

## Results

**Figure 1:**
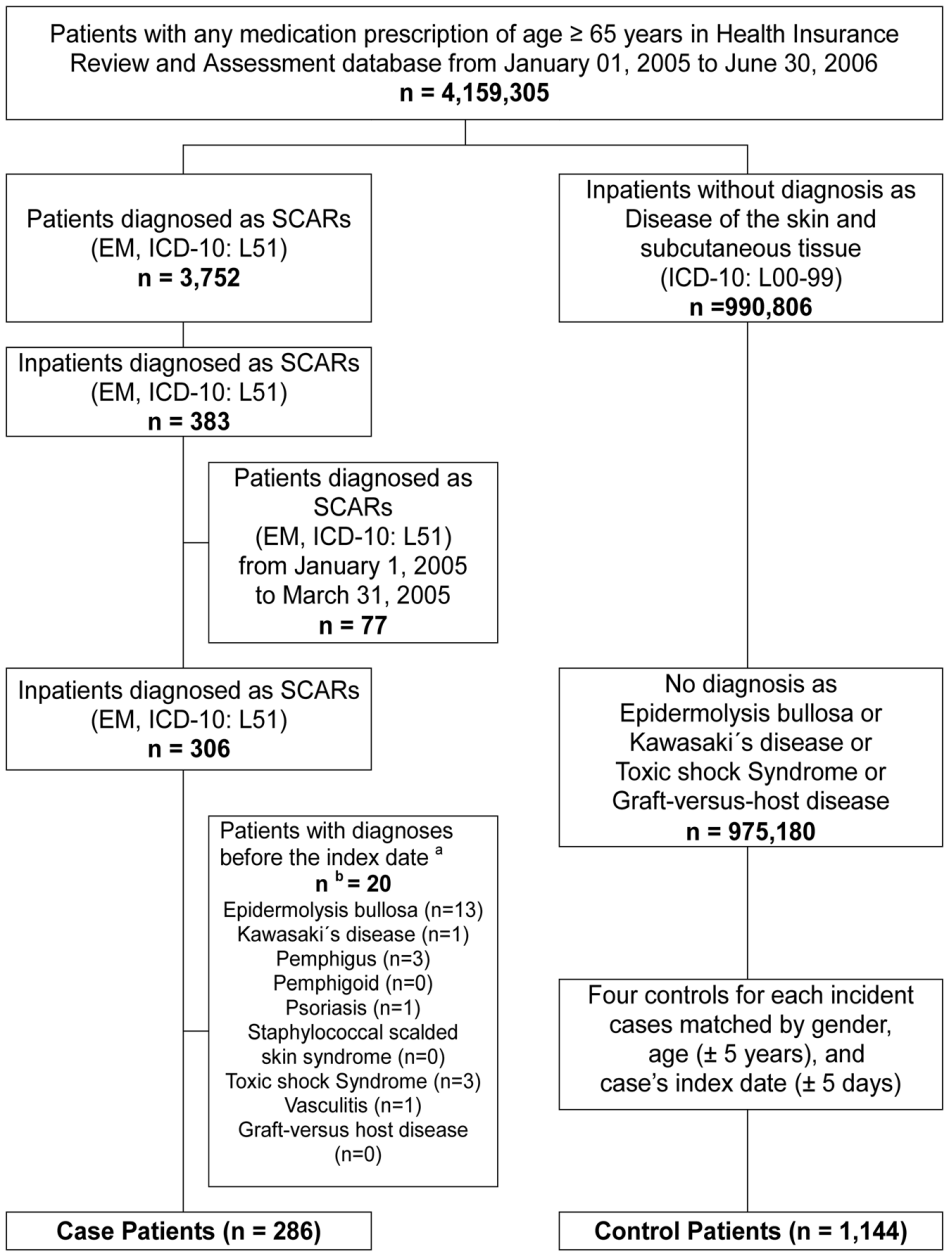
Sampling procedures of cases and controls. Abbreviations; EM, Erythema multiforme; SCARs, Severe cutaneous adverse drug reactions ^a^ Index date: The first SCAR (EM, ICD-10: L51) hospitalization date for the cases. ^b^ The sum of the numbers for the three exclusion criteria is greater than the total number because some patients met more than one exclusion criterion.

A flowchart of the inclusion and exclusion criteria is shown in Figure 1. Our study identified 286 cases and 1,144 matched controls. Table 1 shows the demographic characteristics and use of antiepileptic drugs for both groups before the onset of SCARs. There were 286 cases (121 men and 165 women), with a mean age ± SD of 73.6±6.6 years. Sixty-two (21.7%) cases and 105 (9.2%) controls received at least one antiepileptic drug before the index date. The proportion of antiepileptic drug prescriptions in the cases was significantly higher than that in the controls. The mean Charlson Comorbidity Index score was 2.0 ± 1.5 for cases and 1.4±1.3 for controls, and this difference was statistically significant. Underlying diseases such as cerebrovascular diseases, diabetes mellitus, seizures, and renal diseases were frequent among the cases (*p*<0.001). Similarly, the proportion of patients with connective tissue/rheumatic diseases and herpes virus infection was higher among the cases than the controls. Recent herpes viral infection within 3 months before the index date was reported in only 7 cases and 4 controls. No patients had HIV infection, histoplasmosis, mycoplasma pneumonia, or mumps. There were no statistically significant differences between the two groups in terms of the incidence of other diseases. Eleven (3.8%) cases were diagnosed with severe ocular complications: 2 with corneal ulcer, 2 with corneal ulcers and entropion, 2 with corneal scars and opacities, 2 with entropion, and 3 with visual impairment (table not shown).

**Table 1:** Characteristics of cases and controls (N = 1,430).

	Cases (n = 286)	Controls (n = 1,144)	*P*-value
**Age (years, mean ± SD)**	73.6±6.6	73.6±6.6	1.000
65–69	98 (34.3)	392 (34.3)	
70–74	74 (25.9)	296 (25.8)	
75–79	64 (22.4)	256 (22.4)	
80+	50 (17.5)	200 (17.5)	
**Gender**			1.000
Male	121 (43.3)	484 (43.3)	
Female	165 (57.7)	660 (57.7)	
**Antiepileptic drugs**			<0.001
Yes a	62 (21.7)	105 ( 9.2)	
Mono therapy	45 (15.7)	90 ( 7.9)	
Combination therapy	17 (5.9)	15 ( 1.3)	
No	286 (78.3)	934 (90.8)	
**Charlson Comorbidity Index (mean ± SD)**	2.0±1.5	1.4±1.3	<0.001
0	44 (15.4)	336 (29.4)	<0.001
1	80 (28.0)	357 (31.2)	
2	69 (24.1)	261 (22.8)	
3	45 (15.7)	122 (10.7)	
4 +	48 (16.8)	68 (5.9)	
**Comorbidity** b			
Cancer	31 (10.8)	105 (9.2)	0.392
Cerebrovascular disease	80 (28.0)	151 (13.2)	<0.001
Chronic pulmonary disease	92 (32.2)	323 (28.2)	0.190
Connective tissue/Rheumatic disease	29 (10.1)	55 (4.8)	0.001
Dementia	18 (6.3)	42 (3.7)	0.048
DM without complications	56 (19.6)	123 (10.8)	<0.001
DM with complications	41 (14.3)	104 (9.1)	0.009
Herpes viral infection	7 (2.5)	4 (0.4)	<.0001
Influenza	3 (1.1)	9 (0.8)	0.574
Mild liver disease	38 (13.3)	113 (9.9)	0.093
Myocardial infarction	11 (3.9)	23 (2.0)	0.068
Paraplegia or Hemiplegia	11 (3.9)	22 (1.9)	0.053
Peptic ulcer disease	78 (27.3)	274 (24.0)	0.244
Peripheral vascular disease	19 (6.6)	79 (6.9)	0.875
Renal disease	20 (6.7)	17 (1.5)	<0.001
AIDS/HIV infection	0 (0.0)	0 (0.0)	1.000
Others c	0 (0.0)	0 (0.0)	1.000
**Major indications for antiepileptic drugs**			
Bipolar disorder	1 (0.4)	2 (0.2)	0.563
Diseases of spinal cord	4 (1.4)	6 (0.5)	0.113
Neuropathy	42 (14.7)	122 (10.7)	0.056
Seizure	35 (12.2)	27 (2.4)	<0.001
Trigeminal neuralgia	13 (4.6)	31 (2.7)	0.108

Abbreviations; AIDS/HIV, acquired immune deficiency syndrome/human immunodeficiency virus; DM, diabetes mellitus; SD, standard deviation

Values are presented as number (%).

^a^ Use of all antiepileptic drugs before the index day.

^b^ Patients with more than one underlying disease.

^c^ Other diseases, including histoplasmosis, mycoplasma pneumonia, and mumps.

Table 2 shows the risk estimates of SCARs associated with antiepileptic drugs, calculated by multiple conditional logistic regression analysis. We excluded the antiepileptic drug combination-therapy group, and the final analysis was based on 269 cases matched with 1,076 controls with prescriptions within 60 days of the index date. All antiepileptic drugs showed an increased risk of SCARs (adjusted OR = 3.42; 95% CI: 1.75–6.63). Among the antiepileptic drugs, carbamazepine was associated with the highest risk of SCARs (adjusted OR = 10.39; 95% CI: 2.64–40.86). For gabapentin, phenobarbital, and valproate, the adjusted ORs were 1.10, 2.10, and 8.89, respectively, but no significant association was observed. The risk estimate was consistent when analysis was restricted to patients without recent herpes viral infection. The adjusted OR was 3.69 (95% CI: 1.89–7.23) for all antiepileptic drugs (table not shown).

**Table 2:** Estimates of SCAR risk from antiepileptic drugs within 60 days of index date (N = 1,345).

Antiepileptic drugs	Cases (n = 269)	Controls (n = 1,076)	cOR (95% CI)	aOR b (95% CI)
All †	25 (9.3)	30 (2.8)	3.72 (2.44–8.76)	3.40 (1.76–6.58)
Carbamazepine†	10 (3.7)	4 (0.4)	12.59^a^ (3.22–71.52)	10.69 (2.75–41.57)
Gabapentin	7 (2.6)	22 (2.0)	1.29^a^ (0.46–3.19)	1.13 (0.41–3.17)
Lamotrigine	1 (0.4)	0 (0.0)	4.00^a^ (0.21–∞)	-
Topiramate	0 (0.0)	1 (0.1)	4.00^a^ (0.00–76.00)	-
Phenobarbital	2 (0.3)	3 (0.7)	2.67^a^ (0.22–23.27)	1.79 (0.21–15.49)
Phenytoin	4 (1.5)	0 (0.0)	21.14^a^ (3.59–∞)	-
Valproate	1 (0.4)	1 (0.1)	4.00^a^ (0.05–313.99)	8.89 (0.42–189.92)

Abbreviations; aOR, adjusted odds ratios; cOR, crude odds ratios; SCARs, severe cutaneous adverse drug reactions; 95% CI, 95% confidence interval

Values are presented as number (%).

^a^ Odds ratios calculated by exact conditional logistic regression.

^b^ Odds ratios adjusted for cerebrovascular disease, connective tissue/rheumatic disease, diabetes mellitus, herpes viral infection, seizure, renal disease, Charlson comorbidity index scores, previously highly suspected drugs (allopurinol, aminopenicillins, cephalosporins, imidazoles, macrolides, sulfonamides, tetracyclines and quinolones).

P-value <0.001

**Figure 2:**
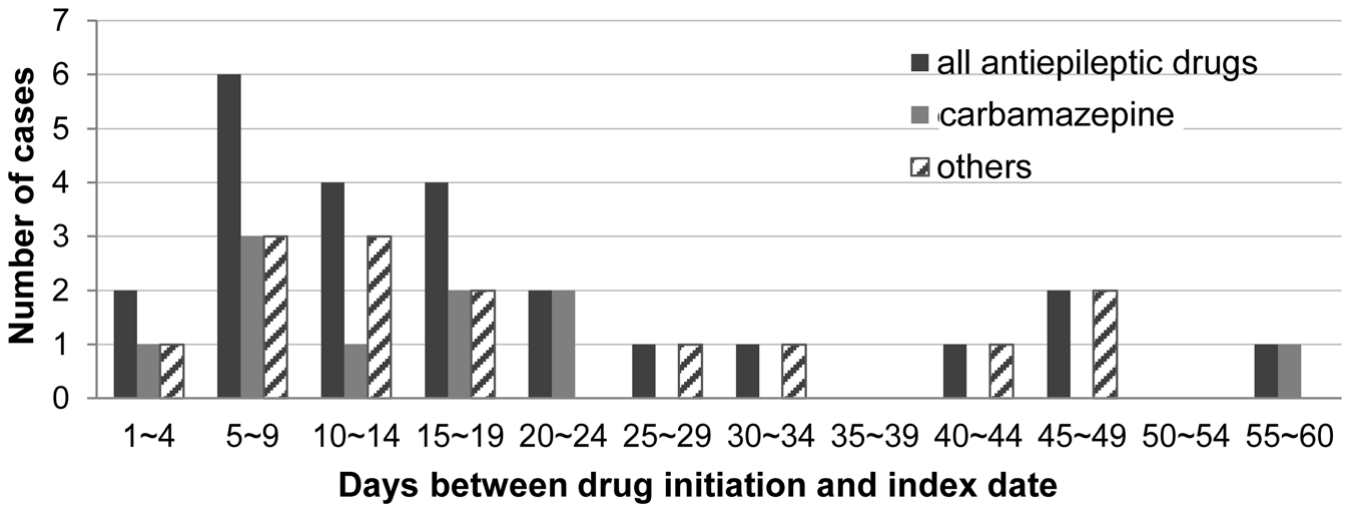
Distribution of latent periods for antiepileptic drugs.

Figure 2 shows the distribution of the latent period in the 25 cases exposed to antiepileptic drugs within 60 days before the index date. Nineteen cases (76%) initiated their treatment less than 30 days before the index date. The median duration of latency (range) for all antiepileptic drugs was 16.0 (range, 3–56) days. The mean duration of prescription for all antiepileptic drugs was 19.3±14.0 days (Table 3). All 25 cases were short-term users (less than 60 days of antiepileptic drugs treatment), and 12 cases (48%) were new users with the first prescription of antiepileptic drugs given during the study period (table not shown).

**Table 3:** Duration of prescription and latent period before onset of SCARs in 25 cases. a

		Prescription duration (days)		Latent period (days)	
Antiepileptic drugs	n	Mean ± SD	Median (Range)	Mean ± SD	Median (Range)
All	25	19.3±14.0	15.0 (3–41)	19.1±15.2	16.0 (3–56)
Carbamazepine	10	20.5±14.3	17.0 (3–41)	17.3±15.4	15.0 (3–56)
Gabapentin	7	11.6±11.4	7.0 (3–31)	23.3±19.3	17.0 (4–49)
Phenobarbital	2	16.0±18.4	16.0 (3–29)	14.0±2.8	14.0 (12–16)
Lamotrigine	1	40.0		17.0	
Phenytoin	4	29.0±10.5	31.5 (15–38)	22.3±16.5	21.0 (5–42)
Valproate	1	8.0		8.0	

Abbreviations; SCARs, severe cutaneous adverse drug reactions; SD, standard deviation

^a^ Cases with prescribed antiepileptic drugs within 60 days of index date.

A subgroup analysis was conducted for 134 cases with SJS or TEN selected using the four-digit ICD-10 codes. Among the 134 cases, 80.6% (n = 108) were classified as having SJS, 17.2% (n = 23) as having TEN, and 2.2% (n = 3) as having both SJS/TEN. The adjusted OR was 10.22 (95% CI: 1.64–63.60) for carbamazepine. The SCAR risk was consistently high in patients treated with carbamazepine for neuropathic pain (adjusted OR = 6.84, 95% CI: 1.55–30.10) (Table 4). Among the 10 cases of carbamazepine use, 8 patients were women; additionally, only 2 of the 10 cases were prescribed carbamazepine for seizure (Table 5).

**Table 4:** Estimates of SCAR risk from carbamazepine.

	Cases	Controls	cOR (95% CI)	aOR b (95% CI)
**Sensitivity analysis**	**n = 269**	**n = 1,076**		
Latent periods less than 30 days†	9 (3.4)	3 (0.3)	12.00^a^ (2.30–68.91)	9.91 (2.48–39.62)
Indications for neuropathic pain†	6 (2.2)	3 (0.3)	8.00^a^ (1.71–49.44)	6.84 (1.55–30.10)
**Subgroup analysis**	**n = 134**	**n = 536**		
Diagnosis with SJS or TEN †	5 (3.7)	3 (0.6)	8.93^a^ (1.43–95.07)	10.22 (1.64–3.60)

Abbreviations; aOR, adjusted odds ratios; cOR, crude odds ratios; SCARs, severe cutaneous adverse drug reactions; SJS, Stevens-Johnson syndrome; TEN, Toxic epidermal necrolysis; 95% CI, 95% confidence interval

^a^ Odds ratios calculated by exact conditional logistic regression.

^b^ Odds ratios adjusted for cerebrovascular disease, connective tissue/rheumatic disease, diabetes mellitus, seizure, renal disease, Charlson comorbidity index scores, previously highly suspected drugs (allopurinol, aminopenicillins, cephalosporins, imidazoles, oxicams NSAIDs, sulfonamides, tetracyclines and quinolones).

*P*-value <0.001.

**Table 5:** Clinical features of 10 cases who prescribed carbamazepine within 60 days of SCARs.

No	Age/gender	Underlying diseases	Indications for CBZ	No of CBZ Prescription	Dose (mg/day)	Duration (days)	Co-medication	Latent period (days)	Acute complications
1	82/Female	HTN, OA	CVD, Trigeminal neuralgia	2	400	8	acetate NSAIDs, aminopenicillins, cephalosporins	3	ND
2	73/Female	HTN	CVD, Trigeminal neuralgia	2	200	38	acetate NSAIDs, oxicams NSAIDs	7	Pneumonia
3	73/Female	Dementia, UTI	ND	2	600	41	aminopenicillins, cephalosporins, sulfonamides	18	ND
4	69/Female	Asthma, HTN, Dementia, MDD, Chronic bronchitis	ND	1	400	11	None	56	Nephritis
5	68/Male	Asthma, COPD	Trigeminal neuralgia	3	400	34	cephalosporins, macrolides	6	ND
6	68/Female	Lumbar disc rupture	CVD, Seizure, Neuropathy	2	400	30	acetate NSAIDs, cephalosporins	23	ND
7	67/Female	Lung cancer, PU	Seizure	1	200	10	acetate NSAIDs, cephalosporins, macrolides	13	Bronchitis
8	67/Female	MDD, OA, Cervical disc disorder	Post-herpetic neuralgia	1	200	23	acetate NSAIDs, aminopenicillins, oxicams NSAIDs	23	ND
9	66/Male	Mild liver disease	Neuropathy	1	200	7	acetate NSAIDs, aminopenicillins, quinolones	7	ND
10	66/Female	Rheumatic disease, DM, PU	Neuropathy	1	200	3	cephalosporins, imidazoles, quinolones	17	Bronchitis

Abbreviations; CBZ, carbamazepine; COPD, chronic obstructive pulmonary diseases; CVD, cerebrovascular disease; DM, diabetes mellitus; HTN, hypertension; MDD, major depressive disorder; ND, not determined; OA, osteoarthritis; PU, peptic ulcer diseases;UTI,urinary tract infection.

## Discussion

In elderly Korean patients, the use of antiepileptic drugs increased the risk of SCARs about three-fold as compared to patients who had not used antiepileptic drugs, and the use of carbamazepine was associated with the greatest increase in the risk of SCARs (adjusted OR = 10.69, 95% CI: 2.75–41.57). Among individual antiepileptic drugs, gabapentin, phenobarbital, and valproate increased the risk of SCARs, but this increase was not statistically significant.

The increased risk of SCARs associated with the use of antiepileptic drugs in the general population has been a consistent finding in several studies [18], [26], [28], [29]. In 2008, one study using the database from Taiwan National Health Insurance showed that the use of carbamazepine and valproate as mood stabilizers increased the risk of SCARs (carbamazepine: adjusted OR = 3.10, 95% CI: 1.51–6.35; valproate: adjusted OR = 5.17, 95% CI: 1.26–5.92). In Taiwan, Gau et al. investigated patients with bipolar disorder only, and the mean age of the subjects was 41 years [18]. Therefore, we cannot make a direct comparison between the results of the study by Gau et al. and our results which focused on elderly patients. In France, the incidence of TEN was 2.7-fold higher among patients aged above 65 years than in those aged below 65 years. This study was performed 20 years ago with a relatively small sample size of only 77 patients [30]. In the pooled analysis of children aged <15 years, Levi et al. found that the crude OR was 91 (95% CI: 14–∞) for all antiepileptic drugs as a group and 15 (95% CI: 1.8–∞) for carbamazepine. However, Levi at al. reported 24 cases and only one control exposed to antiepileptic drugs (total, 80 cases and 216 controls), and therefore, they did not conduct multivariate analysis after adjusting for confounders [29]. According to the SCAR and EuroSCAR studies conducted in European countries, the multivariate relative risk (mRR) of carbamazepine was 12 (95% CI 3.5–38) [26] and 72 (95% CI: 23–225) [28], respectively.

Herpes infection has previously been reported to increase the risk of SCAR in the general population [31], [32]. Our results show significant differences between cases and controls with recent herpes infection. Therefore, we conducted analysis after exclusion of patients with recent herpes infection and the risk of SCAR consistently increased. However, in children, recent herpes infection had no significant association with SCARs [29].

Our results did not find an association between SCARs and lamotrigine, topiramate, phenobarbital, phenytoin, and valproate in the elderly. Because of the small number of patients by each antiepileptic drug, our study did not have enough statistical power to identify certain SCAR risks. However, in the general population, these antiepileptic drugs have frequently been reported to increase the risk of SJS or TEN [1]–[3]. Our study did not show a significantly increased SCARs risk with the use of gabapentin. Gabapentin is usually considered a safe medication for patients with a genetic risk factor of carbamazepine-induced SJS [22] and only a few studies have reported an association between SJS or TEN risk and gabapentin use [33]–[35]. However, in 2011, the US FDA cautioned that potentially serious, multi-organ hypersensitivity, known as drug reaction with eosinophilia and systemic symptoms, has been reported following gabapentin use.

Patients with SJS or TEN frequently had ocular manifestations ranging from conjunctivitis to corneal ulceration [1], which significantly impaired their overall quality of life [36]. Yip et al. reported that 81 (69%) patients with SJS or TEN had ocular complications and 4% had severe ocular involvement during a median follow-up period of 2 months [37]. In our study, only 11 (3.8%) cases diagnosed with severe ocular complications.

Among 25 cases in our study, 13 (52%) patients were prescribed antiepileptic drugs for the first time during the study period. This finding is consistent with previous reports showing an increased risk of antiepileptic drug-induced SCARs, which was mostly observed in short-term or recent-start drug users in the general population [3], [28]. In our study, the mean prescription duration for carbamazepine use was 20.5±14.3 days, which was similar to that for carbamazepine (27.4±14.3 days) in Taiwan [18]. Recently in Korea, Kim et al. reported that the mean treatment duration of carbamazepine was 25.9±15.1 days in 24 carbamazepine-induced SCAR patients [38]. The latent period in our study was defined as the time from the intake of antiepileptic drugs to the first hospitalization due to SCARs, and thus, the mean latent period was 19.1 days for all antiepileptic drug users and 17.3 days for carbamazepine users. The latent period in elderly patients with SCARs was not prolonged as compared to the results of studies in the general population. In the EuroSCAR study, the distribution of the latent period for carbamazepine was similar, ranging from 4 days to 28 days [28]. Yang et al. reported that among 154 SCARs patients, the latent period was 12.32±5.07 days for carbamazepine users and ≤27 days for antiepileptic drug users (lamotrigine, oxcarbazepine, phenobarbital, and phenytoin) [1]. In a recent study based on the EuroSCAR and RegiSCAR databases, Lee et al. showed that prior exposure to corticosteroids prolonged the period of latency before the onset of SJS and TEN [19]. In our analysis, there were only two cases of antecedent corticosteroid users (gabapentin exposure) as per the criteria of Lee et al., and therefore, the result of the latent period was minimally affected. In reality, a mean lag time of 3–3.9 days exists between the date of skin lesion occurrence and the date of hospitalization in children [29]. Therefore, it is reasonable to assume that skin reactions occur 3–4 days before the latent period, and this period can be important for prognosis improvement.

In the present study, a patient admitted to hospital and diagnosed with SJS or TEN with the four-digit ICD-10 codes was considered more valid and severe case, because recording of diagnosis with the four-digit ICD-10 code was not compulsory in Korean clinics during the study period. We applied subgroup analysis for 134 cases with SJS or TEN selected using the four-digit ICD-10 codes, and the results were as follows: for carbamazepine, adjusted OR = 10.22 (95% CI: 1.64–63.60). This result was similar to the result of the initial analysis. Among the 10 cases of carbamazepine use, only 2 patients were prescribed carbamazepine for seizure. Treating pain of neurologic origin was the most common indication of carbamazepine in our study. In Taiwan, peripheral neuropathy was the most frequent underlying disease in antiepileptic drugs-induced SCAR patients [1]. When sensitivity analysis was conducted after selecting cases of carbamazepine use for neuropathic pain, the adjusted OR was 6.84 (95% CI 1.55–30.10). As therapeutic indications have extended, antiepileptic drugs have been used more frequently in pain management [39]. In addition to its use in epilepsy, carbamazepine is also effective in many patients with chronic neuropathic pain [40]. However, carbamazepine is the most common causative agent for SJS or TEN in Southeast Asia, accounting for 26% of cases in Taiwan and 35.7% in Malaysia [41]. Our finding showed that patients who received carbamazepine for pain were consistently associated with risk of SCAR. Physicians should consider medications other than carbamazepine, especially when initiating therapy for pain in elderly patients.

The present study has several limitations. First, lifestyle factors of individual patients, such as smoking, alcohol drinking, family history, and past medical history before the study period were not available. However, the severity of comorbidities was adjusted in the analysis using the Charlson Comorbidity Index score. Moreover, other lifestyle factors are less affected because most SCARs are caused by medication or medical conditions. Second, we used a case-control design in our study. To avoid misclassification bias, the controls were selected from patients who were not hospitalized previously for skin diseases. Lastly, hospital inpatients were selected as controls for each case. Hospital-based controls are unlikely to be representative of the general population and may result in a selection bias because of different hospital admission rates. We compared the admission diagnosis and rate of our study subjects and that of the general population during the study period. The distributions of the admission diagnosis and rate for the controls were consistent with that of the national annual report on elderly patients in Korea.

## Conclusions

In conclusion, the use of carbamazepine increased the risk of severe cutaneous adverse drug reactions by almost 10-fold in Korean elderly patients aged ≥65 years. This association was consistently high with SCAR patients of carbamazepine use for neuropathic pain. When prescribing carbamazepine for the first time to elderly patients, prodromal symptoms that may occur before the skin lesions should be assessed, and special care should be taken to achieve earlier diagnosis and referral in order to improve prognosis.
